# Differences in Survival between Colon and Rectal Cancer from SEER Data

**DOI:** 10.1371/journal.pone.0078709

**Published:** 2013-11-12

**Authors:** Yen-Chien Lee, Yen-Lin Lee, Jen-Pin Chuang, Jenq-Chang Lee

**Affiliations:** 1 Institute of Clinical Medicine, National Cheng Kung University College of Medicine, Tainan, Taiwan, R.O.C; 2 Department of Oncology, Tainan Hospital, Ministry of Health and Welfare, Tainan, Taiwan, R.O.C; 3 Department of Surgery, Tainan Hospital, Ministry of Health and Welfare, Tainan, Taiwan, R.O.C; 4 Department of Surgery, National Cheng Kung University Medical Center, Tainan, Taiwan, R.O.C; Baylor University Medical Center, United States of America

## Abstract

**Background:**

Little is known about colorectal cancer or colon and rectal cancer. Are they the same disease or different diseases?

**Objectives:**

The aim of this epidemiology study was to compare the features of colon and rectal cancer by using recent national cancer surveillance data.

**Design and setting:**

Data included colorectal cancer (1995–2008) from the Surveillance, Epidemiology, and End Results Program (SEER) database. Only adenocarcinoma was included for analysis.

**Patients:**

A total of 372,130 patients with a median follow-up of 32 months were analyzed.

**Main outcome measures:**

Mean survival of patients with the same stage of colon and rectal cancer was evaluated.

**Results:**

Around 35% of patients had stage information. Among them, colon cancer patients had better survival than those with rectal cancer, by a margin of 4 months in stage IIB. In stage IIIC and stage IV, rectal cancer patients had better survival than colon cancer patients, by about 3 months. Stage IIB colorectal cancer patients had a poorer prognosis than those with stage IIIA and IIIB colorectal cancer. After adjustment of age, sex and race, colon cancer patients had better survival than rectal cancer of stage IIB, but in stage IIIC and IV, rectal cancer patients had better survival than colon cancer.

**Limitations:**

The study is limited by its retrospective nature.

**Conclusion:**

This was a population-based study. The prognosis of rectal cancer was not worse than that of colon cancer. Local advanced colorectal cancer had a poorer prognosis than local regional lymph node metastasis. Stage IIB might require more aggressive chemotherapy, and no less than that for stage III.

## Introduction

Colorectal cancer is the third most common cancer among both men and women in the United States [1]. Rectal cancer makes up approximately 25% of large bowel cancers in the Western world. Colon and rectal cancer share many similar clinical features and are often referred to as colorectal cancer. Due to the lack of strong evidence in the setting of rectal cancer, support for the use of adjuvant chemotherapy in patients with rectal cancer is generally extrapolated from the data available for colon cancer [2]. Despite the existence of two entities, 5FU-based chemotherapy regimens are given for both, except radiation therapy is often needed for rectal cancer. Are they the same diseases? If there is really a difference between colon and rectal cancer, we should consider them separately and treat them respectively.

One study tried to answer the question, “Does rectal cancer of the upper third behave more like colon or rectal cancer?” [3]. They concluded that tumor location was an independent prognostic parameter, with an increased risk of cause-specific death for rectal cancers of the upper third and of the middle third, compared to sigmoid cancers. We can at least understand this from the epidemiological study. Therefore, in this population-based study, we compared the prognosis of colon and rectal cancer, using the SEER database.

## Materials and Methods

The SEER program is a population-based cancer registry covering approximately 26% of the US population across several disparate geographic regions and is the largest publicly available cancer dataset. The SEER Registry collects stage at diagnosis, age at diagnosis, cancer type, gender, race. Among deceased persons listed in the SEER Registry, death may have occurred from colorectal cancer or any other cause of death. Using the SEER 1973–2008 database (October 2011 release), we analyzed survival data from all patients diagnosed with colon cancer and rectal cancer for the years 1995–2008. Only the histology of adenocarcinoma was included. For stage, SEER summary stage, which defines stage as localized, regional, or distant were used. The SEER summary stage has been validated, been maintained over time, and correlates well with survival [4].

The anatomic subsites of the proximal colon, distal colon, and rectum were categorized according to the International Classification of Diseases for Oncology, third edition (ICD-0-3) topography codes. The right or proximal colon included cancers of the cecum (ICD-0-3 code C18.0), ascending colon (code C18.2), hepatic flexure (code C18.3), transverse colon (code C18.4), and splenic flexure (code C18.5). The left or distal colon included the descending colon (code C18.6) and the sigmoid colon (code C18.7). Colon cancer also included the large intestine, not otherwise specified (code C18.8, C18.9 and C260). The rectum included the rectosigmoid junction (code C19.9) and the rectum, not otherwise specified (code C20.9).

Overall survival (OS) was determined from the SEER records of survival time and vital satatus. This retrospective population-based study examined whether there was any difference in survival (Death or alive) between colon and rectal cancer. The epidemiologic characteristics of two different locations were described initially.

## Statistical Analysis

Statistical analysis was performed using the SPSS 13.0 statistical package. Patient characteristics were described using summary statistics. *P*-values for comparing these patient characteristics between the colon and rectal cancer were calculated using chi-squared test. Two sample T test was used to compare age. Kaplan-Meier and the Cox proportional hazard models were used to compare overall survival. Log-rank *p*-values based on the Cox proportional hazard models were used to compare the survival and cumulative event curves. Length of survival was later calculated from the date of diagnosis until either the time of death or the end of follow-up. All tests were two-sided, and a *P* value of <.05 was considered statistically significant.

## Results

### Patient Characteristics

The study group consisted of 372,130 patients with a median follow-up of 32 months (range, 0–167 months; interquartile range, 11–68 months), and included 192,810 men (51.8%) and 179,320 women (48.2%). The median age was 71 years (range, 9–110 years). A total of 261,523 patients (70.3%) had colon cancer, and 110,607 (29.7%) had rectal cancer. The colon cancer incidence was twice greater than the rectal cancer incidence in the current cohort (Table 1). The median age of the rectal cancer patients at diagnosis was 5 years less than that of the colon cancer patients. Median age of the colon cancer patients was 72 years (interquartile range, 61–80) and that of the rectal cancer patients was 67 years (interquartile range, 56–76). Male and female had reverse proportion of colon and rectal cancer. Females accounted for 50.3% of the colon cancer group and 43.2% of the rectal cancer group. In terms of location of adenocarcinoma of the colon, the sigmoid accounted for 23.1%, followed by the cecum 15.6% and the ascending colon 12%; 27.6% were right-sided (cecum, ascending colon) and 57.3% were left-sided (descending, sigmoid, rectosigmoid, rectum) tumors (Table 2).

**Table 1:** Characteristics of all subjects.

	Colon cancer	Rectal cancer	P Value
Total Number	261,523	110,607	
Age, years (IQR)	72 (61–80)	67 (56–76)	<0.001*
range	(9–110)	(13–107)	
Female, N (%)	131,554 (50.3)	47,766 (43.2)	<0.001*
Ethnicity, N (%)			
White	212,446 (81.2)	89,779 (81.2)	0.646
Black	28,062 (10.7)	9,466(8.6)	<0.001*
Others	21,015 (8.1)	11,362 (10.2)	<0.001*
Stage, N (%)	95,087 (36.4)	36,567 (33.1)	
I	25,151 (9.6)	11,798 (10.7)	
II	26,976 (10.3)	8280 (7.5)	
III	24,748 (9.5)	9,773 (8.8)	
IV	18,212 (7)	6,716 (6.1)	

All values are median (interquartile range) or N (%),

* *p*<0.05.

**Table 2:** Distribution of colorectal adenocarcinoma among the different sites.

	Number	N (%)	Cumulative N (%)
Cecum	58,181	15.6	15.6
Ascending colon	44,779	12.0	27.7
Hepatic flexure	13,880	3.7	31.4
Transverse colon	23,751	6.4	37.8
Splenic flexture	9569	2.6	40.4
Descending colon	16,920	4.5	44.9
Sigmoid colon	85,912	23.1	68
Large intestine, not otherwise specified	8531	2.2	70.2
Rectosigmoid junction	32,305	8.7	78.9
Rectum	78,302	21.0	100

### Survival

**Figure 1:**
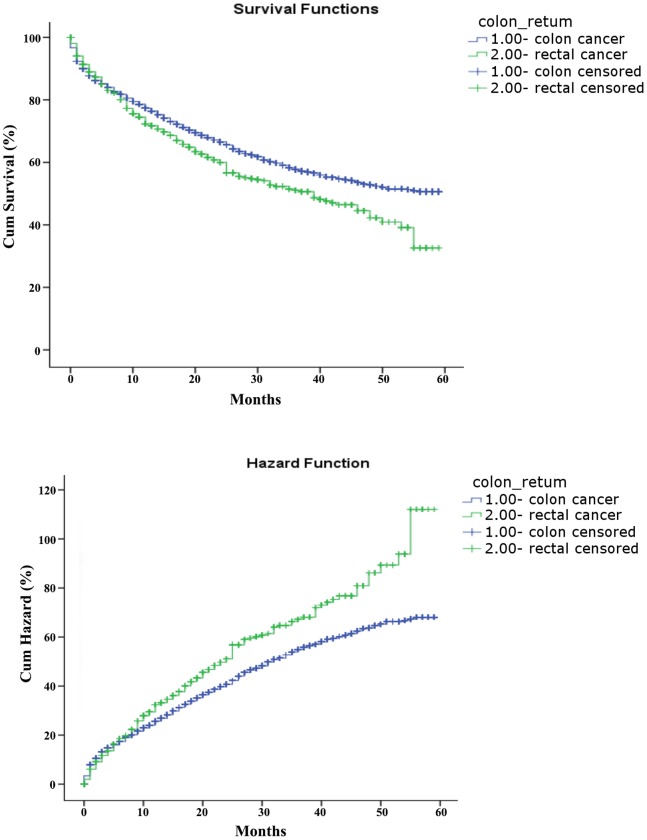
Survival and cumulative hazard for stage IIB colon and rectal cancer patients (1, colon cancer; 2, rectal cancer).

**Figure 2:**
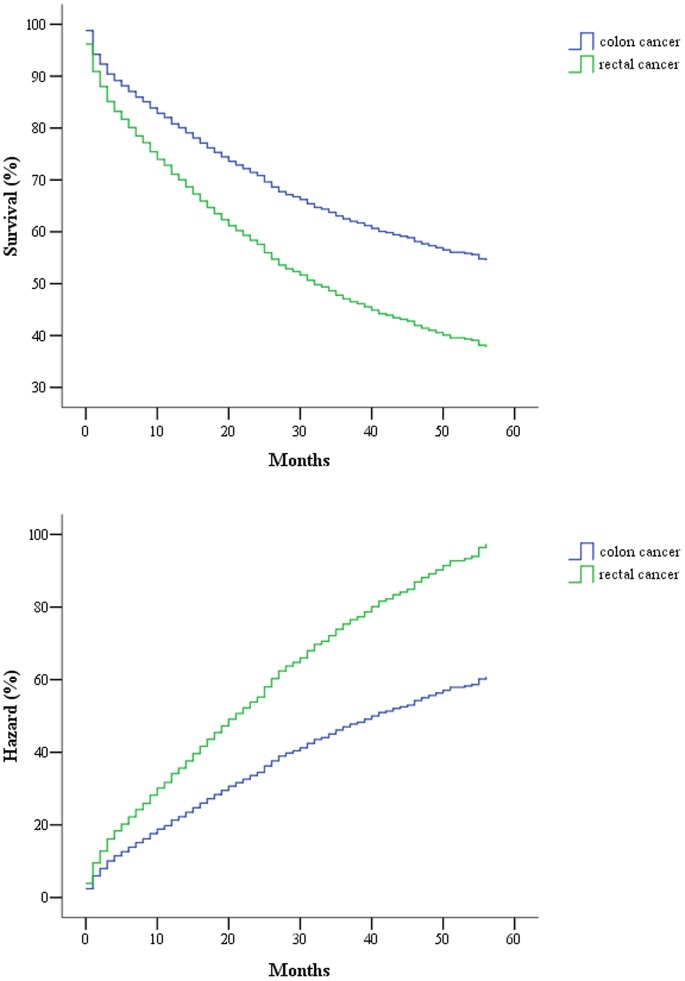
Survival and cumulative hazard in Cox regression model of stage IIB colon and rectal cancer patients.

**Figure 3:**
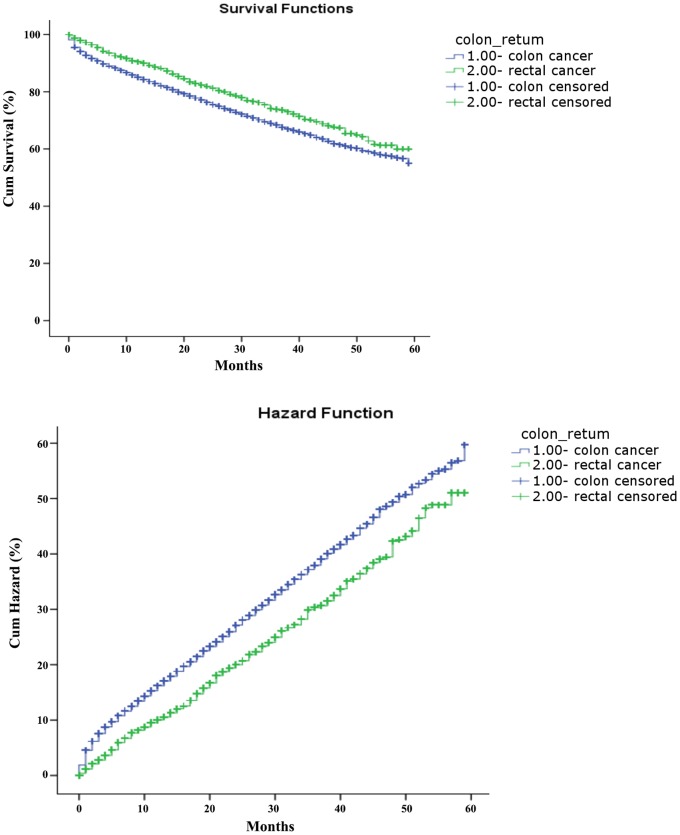
Survival and cumulative hazard of stage IIIB colon and rectal cancer patients (1, colon cancer; 2, rectal cancer).

**Figure 4:**
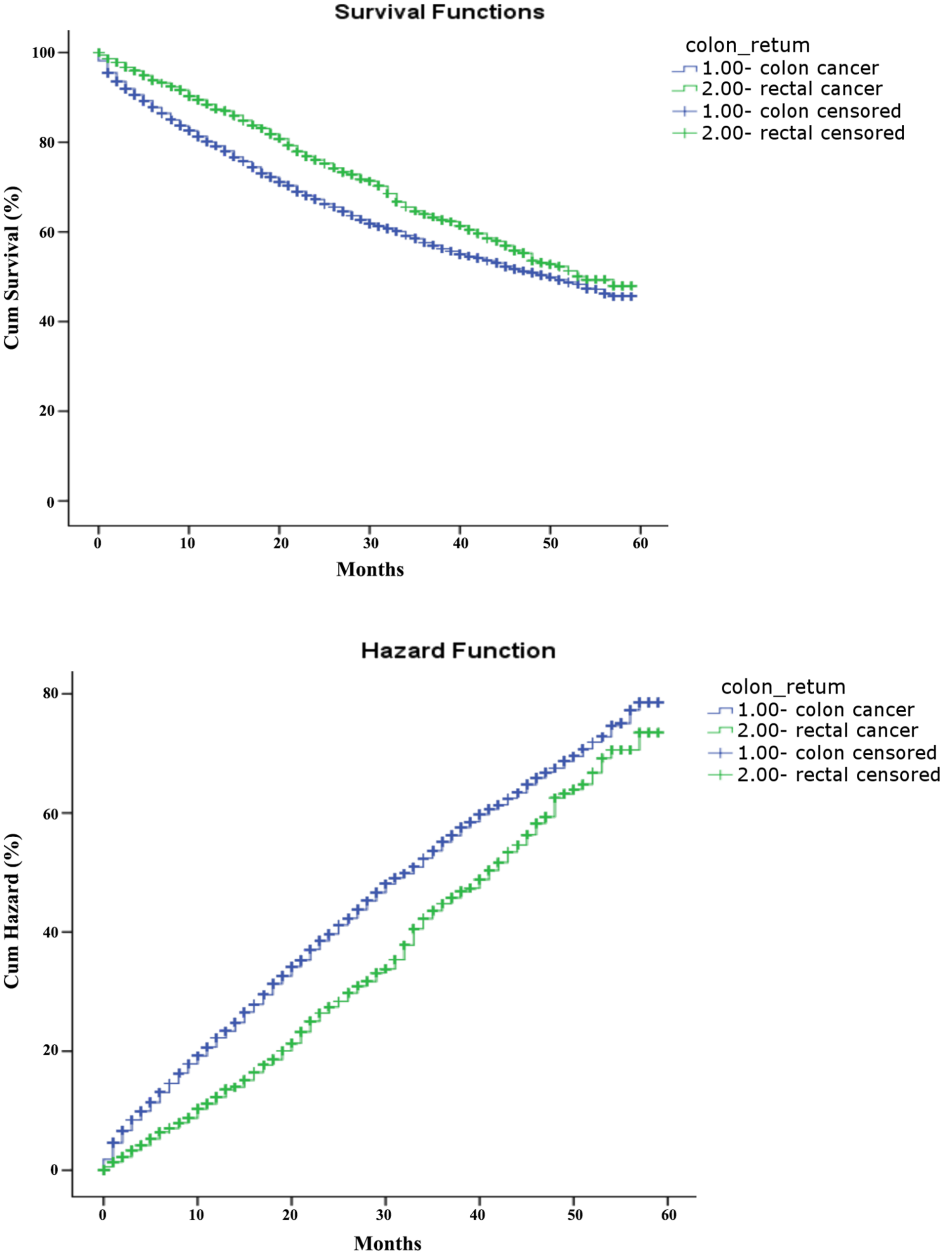
Survival and cumulative hazard of stage IIIC colon and rectal cancer patients (1, colon cancer; 2, rectal cancer).

**Figure 5:**
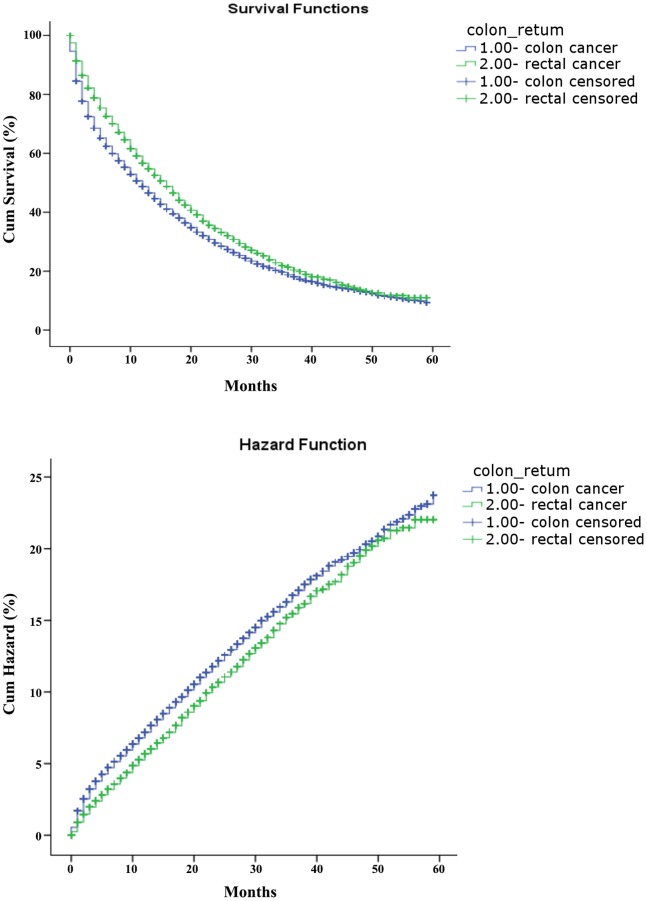
Survival and cumulative hazard of stage IV colon and rectal cancer patients (1, colon cancer; 2, rectal cancer).

**Figure 6:**
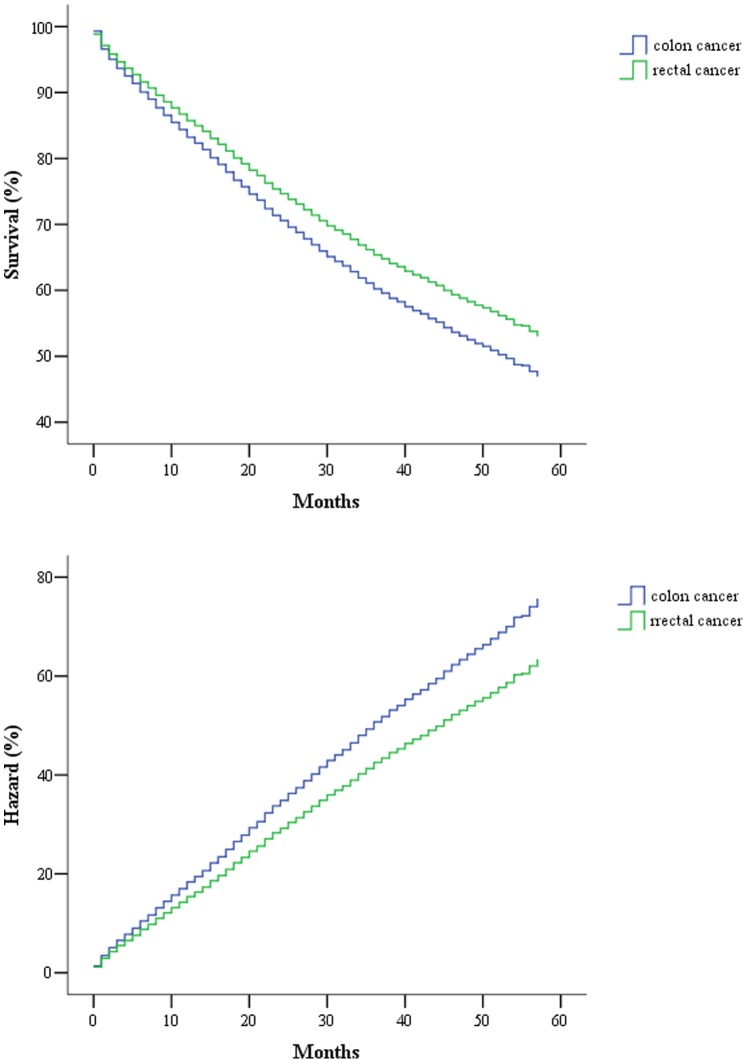
Survival and cumulative hazard in Cox regression model of stage IIIC colon and rectal cancer patients.

**Figure 7:**
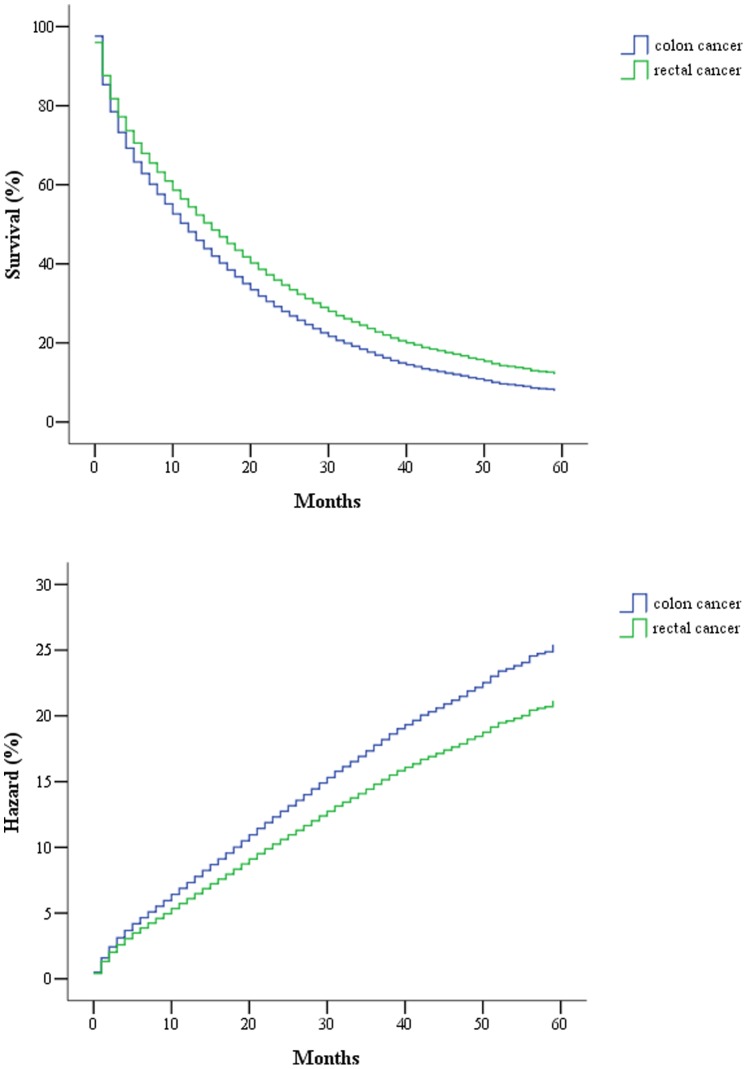
Survival and cumulative hazard in Cox regression model of stage IV colon and rectal cancer patients.

Only about 40% of the SEER data during this period had stage information. There is no difference in characteristics between those with stage and without stage information (Table 3). The stages were roughly equally distributed. In the survival analysis for stages I, IIA, and IIIA, there were no differences between colon and rectal cancer. As for stage IIB, patients with colon cancer had a 4-month longer survival than those with rectal cancer (Table 4, Figure 1). After adjustment of age, sex and race, there is still a difference in survival between colon and rectal cancer (Table 5, Figure 2). In stages IIIB, IIIC, and IV, patients with rectal cancer seemed to have a better survival range (Table 4, Figure 3, 4, 5), from 1 to 4 months longer than those with colon cancer. In stage IIIB, sex, race and age accounted for the difference. In stage IIIC and IV, after adjustment for sex, race and age, there are still difference in colon and rectal cancer (Table 5, figure 6, 7). Of note, stages IIIA and IIIB patients seemed to have better survival than those with stage IIB (Table 4).

**Table 3:** Characteristics of the subjects with and without stage information.

Stage information	Colon cancer		Rectal cancer	
	Yes	No	Yes	No
Total Number	111,825	149,698	47,841	62,766
Age, years (IQR)	71 (60–80)	72 (62–80)	65 (54–76)	68 (57–77)
Female, N (%)	56,023 (50.1)	75,531 (50.5)	20,509 (42.9)	27,257 (43.4)
Ethnicity, N (%)				
White	89,924 (80.4)	122,522 (81.8)	38,250 (80.0)	5,124 (82.1)
Black	12,439 (11.1)	15,623 (10.4)	4,342 (9.1)	5,124 (8.2)
Others	9,462 (8.5)	11,553 (7.8)	5,249 (10.9)	6,113 (9.7)

All values are median (interquartile range) or N (%).

**Table 4:** Survival of patients (year) with different stages of colon or rectal cancer by Kaplan-Meier.

	Colon cancer		Rectal cancer		
Stage	No.	mean survival (95% CI)	No.	mean survival (95% CI)	p value
I	28, 100	49.873 (39.627–50.119)	8847	49.531 (49.091–49.970)	0.247
IIA	25,932	47.681 (47.408–47.955)	4922	47.392 (46.760–48.025)	0.569
IIB	3775	38.639 (37.784 –39.495)	627	34.604 (32.493–36.714)	0.001*
IIIA	3388	50.350 (49.662–51.037)	1156	50.667 (49.509–51.825)	0.538
IIIB	15,264	45.570 (43.177–43.962)	3549	46.526 (45.763–47.288)	<0.001*
IIIC	9297	38.864 (38.323–39.406)	1755	42.566 (41.413–43.720)	<0.001*
IV	20,503	18.743 (18.440–19.046)	4425	21.341 (20.677–22.005)	<0.001*

* *p*<0.05.

**Table 5:** Cox’s regression for colorectal cancer.

	Hazardratio	95% CI forHazard ratio	*P*-value
**Stage I**			
Colon vs. rectal cancer	0.996	0.942–1.054	0.900
Sex	1.094	1.038–1.152	<0.001*
Race	2.111	1.830–2.435	<0.001*
Age	1.009	1.009–1.009	<0.001*
**Stage IIA**			
Colon vs. rectal cancer	0.997	0.939–1.059	0.933
Sex	1.094	1.039–1.151	0.026*
Race	1.882	1.638–2.164	<0.001*
Age	1.008	1.007–1.008	<0.001*
**Stage IIB**			
Colon vs. rectal cancer	0.624	0.556–0.700	<0.001*
Sex	1.201	1.085–1.329	<0.001*
Race	1.730	1.365–2.194	<0.001*
Age	1.045	1.045–1.050	<0.001*
**Stage IIIA**			
Colon vs. rectal cancer	0.961	0.814–1.133	0.633
Sex	1.228	1.052–1.432	0.009*
Race	1.393	1.972–1.995	0.071
Age	1.074	1.066–1.082	0.004*
**Stage IIIB**			
Colon vs. rectal cancer	0.998	0.931–1.069	0.947
Sex	<0.001	1.068–1.199	<0.001*
Race	<0.001	1.376–1.818	<0.001*
Age	0.001	1.053–1.058	0.001*
**Stage IIIC**			
Colon vs. rectal cancer	1.193	1.100–1.295	<0.001*
Sex	1.046	0.979–1.117	0.187
Race	1.869	1.592–1.592	<0.001*
Age	1.048	1.045–1.050	<0.001*
**Stage IV**			
Colon vs. rectal cancer	1.202	1.160–1.245	<0.001*
Sex	0.968	0.939–0.998	0.037*
Race	1.335	1.245–1.431	<0.001*
Age	<0.001	1.008–1.008	<0.001*

adjust for sex, race (white, black and others), age;

* *p*<0.05.

## Discussion

Though AJCC Cancer Staging Handbook [5] have already reported survival data on colon and rectal cancers separately, we are the first group to compare them together in this epidemiology study. The incidence of colon cancer was twice higher than that of rectal cancer in this cohort study. White people had an 8 times higher incidence than black people, which was roughly proportional to the racial distribution of the United States. Women had higher proportion of colon cancer than rectal cancer while compared with men. The gender ratio was reverse between colon and rectal cancer. Due to unable to obtain statistic significant of median survival with stage stratification between colon and rectal cancer (data not shown), only mean survival was reported in Table 4. Owing to large population here, we believe that it is unlikely to reach any significant median value of survival between colon and rectal cancer in the near future. For the adenocarcinoma histology in the 1995 to 2008 SEER database, after adjusted for age, sex and race, colon cancer patients had better survival in the early stage (stage IIB). However, in the more advanced later stages, stages IIIC and IV, rectal cancer patients seemed to have longer survival than colon cancer patients after adjustment. This survival disparity may be explained by different underlying genetic factors or differences in the blood supply direction or the metastasis direction besides sex, race and age, though this is still remains unknown. Stages IIIA and IIIB seemed to have better survival than stage IIB. This might indicate that when considering the prognosis, local factors might not be less important than local lymph node metastasis.

Colon and rectal cancer share many features and are often referred to as colorectal cancer. Some studies grouped them together [6], [7] and some did not. As for dietary factors, methionine was associated with a decreased risk of proximal colon cancer among men and rectal cancer among women [8], while other reported only protective in rectal cancer [9]. Vitamin B-6 was positively associated with rectal cancer but protective in both colon and rectal cancer in another [10], [11]. One meta-analysis showed that vitamin D decreased both colon and rectal cancer [8], [12]. Increasing intakes of calcium and insoluble dietary fiber have been associated with a decreasing risk of colon cancer. Carbohydrate intake was positively correlated with the risk of rectal cancer and fat consumption was inversely correlated with the risk of female colon and rectal cancers [13]. Consumption of red meat and processed meat was positively associated with risk of both colon and rectal cancer, with stronger association with red meat for rectal cancer [14], [15]. Increased physical activity may decrease the risk of colon cancer, but not rectal cancer in two meta-analysis [16], [17]. Heavy smokers have been associated both with rectal cancer (stronger) and colon cancer in two meta-analysis [18], [19] and one pooled analysis [20]. Proximal colon cancers are more likely than rectal and distal colon tumors to have microsatellite instability, a CpG island methylator phenotype, and KRAS mutations, whereas rectal and distal colon tumors are more likely than proximal colon tumors to have a p53 mutation [21]. There was also a difference in protein expression and gene amplification of cyclins between colon and rectal adenocarcinoma [22]. On contrary, one paper reported that excluding the hypermutated cancers, colon and rectum cancers were similar in genomic alteration [23]. Even in colon cancer, there are still differences in gene expression between normal mucosa and the adenocarcinomas, and between adenocarcinomas of the cecum and sigmoid or rectosigmoid [24]. In clinical specimens, approximately 30–50% of colon cancers were reported to harbor KRAS mutations [25], [26]. Codon 12 mutations were also associated with a poor prognosis in colon cancer [27]. Of 57 rectal cancer patients in one study, 31.6% carried mutations in KRAS genes, and 9.6% had a loss of PTEN expression with no detected BRAF mutations [28]. In a study of 96 locally advanced rectal cancer patients undergoing neoadjuvant chemoradiation therapy, 38% had KRAS mutations. KRAS mutations were found in 15% of 134 Finnish women [29]. One study reported that KRAS mutation status was not related to outcomes in rectal cancer [30], and another study showed a contrary result [31]. High levels of microsatellite instability have been associated with an improved prognosis in colon cancer and with a poor prognosis in rectal cancer [32].

Human colon cancer tissues were reported to be more sensitive than rectal cancer tissues to antitumor drugs *in vitro* [33]. However, in our analysis, advanced stage rectal cancer patients had a 3–4-month better overall survival than colon cancer patients. Gene expression profiles and carcinogenesis pathways have been shown to differ between colon and rectal cancer, with metabolic pathways being more important in rectal cancer. The oncogenesis of rectal cancer may be more complex than that of colon cancer [34]. A study from a single institute reported that the prognosis of colon cancer was significantly better than that of rectal cancer, especially for stage III [35]. Our study results, with a larger and multicenter population, showed that advanced stage rectal cancer patients had better overall survival. Another study reported that 5-year survival for patients with colonic tumors was 76%, and for rectal tumors was 69%. The difference was attributed to a higher proportion of Dukes’ stage C tumors in the rectum and better survival prospects for patients with colonic rather than rectal stage C1 tumors [36]. Thirty years have passed, and Dukes’ stage C1 colon cancer patients still had better overall survival than patients with rectal stage C1 tumors in our study.

Our results also showed that right-sided (cecum, ascending colon) and left-sided (descending, sigmoid, rectosigmoidal, rectum) tumors accounted for 27.6% and 57.3% of colorectal adenocarcinoma, respectively. Sigmoidoscopy screening could detect approximately 52.8% of large intestinal cancers.

There are several limitations of SEER databases during this period. 40% of SEER databases during this period had stage information. Also, only about 12.7% surgery-related information could be obtained for this group of patients. Complete chemotherapy and radiation therapy information couldn’t be obtained as well. Data from the SEER cancer registries and the Medicare claims files of the Health Care Financing Administration have to be linked in order to study the proportion of those who had received chemotherapy and radiation therapy. The Medicare program only provides health insurance for 97% of the United States population aged 65 and older. Even while linking to the Medicare system, only patient aged 65 and older could be obtained. We consider our study population is larger. Regarding to overall survival, this is more closely to the true world.

### Conclusions

To our knowledge, this is the first innovation paper to compare colon and rectum cancer from epidemiology data and there are differences between colon and rectal cancer survival and characteristics. More histological or genetic studies are needed for more detailed clarification.
